# *OsSPL10*, a SBP-Box Gene, Plays a Dual Role in Salt Tolerance and Trichome Formation in Rice (*Oryza sativa* L.)

**DOI:** 10.1534/g3.119.400700

**Published:** 2019-10-05

**Authors:** Tao Lan, Yali Zheng, Zilong Su, Shibo Yu, Haibing Song, Xiaoya Zheng, Gege Lin, Weiren Wu

**Affiliations:** * Key Laboratory of Genetics, Breeding and Multiple Utilization of Crops, Ministry of Education,; † Key Laboratory of Applied Genetics of Universities in Fujian Province, and; ‡ Fujian Provincial Key Laboratory of Crop Breeding by Design, Fujian Agriculture and Forestry University, Fuzhou, Fujian 350002, China

**Keywords:** Rice, *SST*, SBP-box, Salt tolerance, Glabrous leaf and glume

## Abstract

Salinity is one of the major abiotic stress factors limiting rice production. Glabrousness is a trait of agronomic importance in rice (*Oryza sativa* L.). We previously found a single-gene recessive mutant *sst*, which displayed increased salt tolerance and glabrous leaf and glume without trichomes, and identified an SBP-box gene *OsSPL10* as the candidate of the *SST* gene. In this study, *OsSPL10*-knockout and *OsSPL10*-overexpression mutants were created to check the function of the gene. The knockout mutants exhibited enhanced salt tolerance and glabrous leaves and glumes as expected, while the overexpression mutants showed opposite phenotypes, in which both salt sensitivity and trichome density on leaf and glume were increased. These results clearly confirmed that *OsSPL10* is *SST*, and suggested that *OsSPL10* controls the initiation rather than the elongation of trichomes. In addition, expression analysis indicated that *OsSPL10* was preferentially expressed in young panicle and stem, and protein OsSPL10 was localized in nucleus. Taken together, *OsSPL10* negatively controls salt tolerance but positively controls trichome formation in rice.

Rice is sensitive to salt stress, which suppresses rice growth and development, causing severe yield loss. Salinity tolerance is a quantitative trait controlled by multiple genes in rice (Ren *et al.* 2005). Salt tolerance mechanism is complex in rice, involving many pathways. Certain apoplastic proteins are involved in the initial phase of salt stress response (Zhang *et al.* 2009). Some receptor-like kinases (RLKs) mediate salt sensitivity or improve salt tolerance by regulating ethylene homeostasis or H_2_O_2_ homeostasis (Li *et al.* 2014; Zhou *et al.* 2018). G-protein and small G-protein also play roles in salt-induced cellular senescence and other salt sensitivity (Urano *et al.* 2014; Zang *et al.* 2010). Calcineurin B-like protein S (CBLs), CBL-interacting protein kinases (CIPKs), and calcium-dependent protein kinases (CPKs) function in salt signal transduction (Martínez-Atienza *et al.* 2007; Campo *et al.* 2014). Some cation transporters of plasma membrane, such as *OsSOS1*, *OsHKT1;5* (*SKC1*), *OsKAT1*, *OsHAK1*, and *OsMGT1*, which are sodium, potassium and magnesium transporter (or channel), respectively, are related to salt tolerance (Martínez-Atienza *et al.* 2007; Ren *et al.* 2005; Obata *et al.* 2007; Chen *et al.* 2015; Chen *et al.* 2017). Many different transcription factors are involved in salinity tolerance or sensitivity, including NAC, OsbZIP23, DST, OsWRKY45-2, DREB1B, OsMYB2, SERF1 and so on (Hu *et al.* 2006; Xiang *et al.* 2008; Huang *et al.* 2009; Tao *et al.* 2011; Datta *et al.* 2012; Yang *et al.* 2012; Schmidt *et al.* 2013). In addition, epigenetics is also involved in salt tolerance in rice (Yuan *et al.* 2015; Srivastava *et al.* 2016; Wang *et al.* 2017).

Glabrous rice varieties have glabrous leaves and glumes without trichomes. In most terrestrial plants, trichomes are specialized structures, which originate from the above-ground epidermal tissues and develop into hair-like projections extending from the epidermal surfaces through growth, differentiation or cell division (Johnson 1975). As trichomes can lead to the generation of dust during harvesting and grain manipulating processes in rice production, glabrous leaves and glumes are a desirable characteristic. Only two glabrous genes have been cloned in rice so far, namely, *OsWOX3B* (*DEP*, *NUDA*/*GL-1*, *GLR1*) and *OsPLT2* (*HL6*). The former is a *WUSCHEL*-like homeobox gene (Angeles-Shim *et al.* 2012; Zhang *et al.* 2012; Li *et al.* 2012), while the latter encodes an AP2/ERF transcription factor, which physically interacts with OsWOX3B (Sun *et al.* 2017).

SQUAMOSA Promoter-Binding Protein (SBP) and SBP-Like (SPL) proteins are putative transcription factors, which have a plant-specific SBP domain consisting of 76 amino acids in length (Cardon *et al.* 1997). *SBP* genes (*SBP1* and *SBP2*) were first isolated from *Antirrhinum majus* and found to control early flower development by regulating the MADS-box gene *SQUAMOSA* (Klein *et al.* 1996). Then, *SPL3* involved in floral transition was isolated from *Arabidopsis thaliana* (Cardon *et al.* 1997) and LG1 with SBP domain was found to be required for induction of ligules and auricles during maize leaf organogenesis (Moreno *et al.* 1997). *SPL* gene family is not large, with only 17 members in *Arabidopsis* and 19 in rice (Xie *et al.* 2006). *SPL* genes have been shown to play numerous important roles in plant growth and development, including trichome development and fertility (Unte *et al.* 2003; Xing *et al.* 2010), lateral root development (Yu *et al.* 2015), fruit ripening (Manning *et al.* 2006), plastochron length, flowering pathway and organ size (Wang *et al.* 2008, 2009; He *et al.* 2018), yield (Chuck *et al.* 2014; Si *et al.* 2016; Zhang *et al.* 2017), copper homeostasis (Yamasaki *et al.* 2009; Yan *et al.* 2017), and so on. Some *SPL* genes are related to abiotic stress tolerance. SPL1 and SPL12 confer thermotolerance at reproductive stage in *Arabidopsis* (Chao *et al.* 2017). Down-regulation of *MsSPL8* leads to enhanced salt and drought tolerance in alfalfa (Gou *et al.* 2018).

In rice, it has been found that *OsSPL* genes control a large range of processes underlying plant growth and development (Wang and Zhang 2017). For example, *OsSPL8* (*OsLG1*) controls ligule development and inflorescence architecture (Lee *et al.* 2007; Ishii *et al.* 2013; Zhu *et al.* 2013). *OsSPL13* (*GLW7*) controls grain size (Si *et al.* 2016). *OsSPL14* (*IPA1*, *WFP*) affects tiller number and panicle branching (Jiao *et al.* 2010; Miura *et al.* 2010) and promotes immunity (Wang *et al.* 2018). *OsSPL16* controls grain size, shape and quality (Wang *et al.* 2012) and plays a role in panicle cell death during ER stress (Wang *et al.* 2018). *OsSPL18* controls grain weight and grain number in rice (Yuan *et al.* 2019).

We previously obtained a rice mutant *sst* showing salt tolerance and glabrous leaves and glumes from a restorer line R401. We found that *sst* was controlled by a recessive gene, which was likely to result from a deletion of one nucleotide in *OsSPL10* (LOC_Os06g44860, Os06g0659100), an SBP-box gene (Wang *et al.* 2013; Lan *et al.* 2015; Song *et al.* 2016). In this study, we confirmed the function of *OsSPL10* as the candidate of *SST* through gene knockout and overexpression, investigated the expression pattern of *OsSPL10*, and analyzed the subcellular localization of OsSPL10, aiming to lay a foundation for deep studies of the molecular mechanism of *OsSPL10* function in salt tolerance and trichome development.

## Materials and Methods

### Plant materials

The following plant materials were used or created in this study: *indica* rice cultivars R401 and Huanghuazhan (HHZ); *japonica* rice cultivars Nipponbare and Zhonghua 11 (ZH11); the salt-tolerant and glabrous-leaf mutant *sst* obtained from R401 by radiation mutagenesis (Wang *et al.* 2013; Lan *et al.* 2015; Song *et al.* 2016); *OsSPL10*-knockout mutant plants from HHZ and ZH11; and *OsSPL10*-overexpression plants from ZH11. All rice plants were grown in plastic trays with paddy soil under a long day condition (approximately 14 h light/day) in the growth chamber (with cool-white light 300 μmol m^2^ s^-1^).

### Knockout of OsSPL10

The CRISPR/Cas9 editing system were used to knock out *OsSPL10* in ZH11 and HHZ. Two target sites (5′-GTTCGGGGCGATGCAGGCG-3′ and 5′-CACCACCACCACATGTACGA-3′) upstream to the *sst* mutation site in the first exon of *OsSPL10* were selected and isolated according to the rules of low off-target score and high sgRNA score (http://cbi.hzau.edu.cn/crispr/), and then inserted into the VK005-01 binary vector containing the rice U6 promoter (Viewsolid Biotechnology Company of Beijing). The construct was introduced into ZH11 and HHZ using the stable transformation method (Hiei and Komari 2008). To examine mutations occurred in positive transgenic (T_0_) plants, a 400-bp genomic DNA fragment harboring the two target sites was amplified from them by PCR using primers 5′-AGCTCCACTTCGTTGGAAGCCA-3′ and 5′-GCGACACGCTGTAGCACGGT-3′ and then sequenced. Homozygous mutants obtained in T_1_ generation were phenotyped for salt tolerance and trichomes on leaves and glumes.

### Overexpression of OsSPL10

Total RNA was extracted from the young panicles (<5 cm) of Nipponbare and converted into cDNA by reverse transcription. RNA extraction was performed using TRIzol reagent (Invitrogen, USA). PrimeScriptTM RT reagent Kit (Takara, Japan) was used to synthesize the first strand of cDNA with OligodT primer. The 1.2-kb coding sequence of *OsSPL10* was amplified from the cDNA by PCR using primers 5′-ATGATGAGCGGTAGGATGAA-3′ and 5′-CTACATGAAGTCGACCTCGA-3′, and then inserted into the pCXUN vector containing the maize *ubiquitin* promoter. The construct was introduced into ZH11 using the stable transformation method (Hiei and Komari 2008). The positive transgenic plants overexpressing *OsSPL10* were phenotyped for salt tolerance and trichomes on leaves and glumes.

### Measurement of salt tolerance

Rice seeds were sown on paddy soil in plastic trays (36×28×4.5 cm^3^) after pregermination and allowed to grow at 26° under a photoperiod of 14 h light/10 h dark in a growth chamber. Salt stress treatment began from late two-leaf stage. During the treatment, 200 mL of either NaCl solution (150 mM) or fresh water was added into each tray every day. The treatment procedure for the *OsSPL10*-knockout seedlings was: 7 d NaCl → 3 d water → 7 d NaCl → 3 d water, while that for the *OsSPL10*-overexpression seedlings was: 7 d NaCl → 3 d water → 4 d NaCl. The survival rate of seedlings was investigated at the end of the treatment.

### Measurement of leaf trichome density

The penultimate leaves of individual plants were collected at tillering stage or heading stage. The adaxial surface of the middle part of each leaf was observed with scanning electron microscopy (SEM). The number of trichomes within a field of vision was counted, and three fields of vision were investigated on each leaf.

### Quantitative RT-PCR of OsSPL10

Total RNA was extracted from seedlings as well as flag leaf blades, flag leaf sheaths, mature (second) leaf blades, mature leaf sheaths, stems, pre-emergence inflorescences and young panicles at the booting stage. RNA extraction and cDNA synthesis were conducted using the same methods as described above. The qRT-PCR was performed using SYBR Premix Ex TaqTM (Tli RNaseH Plus) (Takara, Japan) on a Prism 7500 96 Real-time PCR System (ABI, USA). The primers for *OsSPL10* were 5′-ACAACGACAACAGCCACAACAA-3′ and 5′-ACACGAACACATGGTAGGATCGA-3′. The *actin* mRNA level was used as internal reference, for which the primers were 5′-AGTGCGACGTGGATATTAGG-3′ and 5′-TGGCTTAGCATTCTTGGGT-3′. Three independent biological replicates were analyzed by qRT-PCR in triplicate. The changes in gene expression were calculated using the 2^-ΔΔCt^ method.

### Subcellular localization of OsSPL10

*GFP* cDNA was fused to the C-terminus of *OsSPL10* cDNA (without terminator) in the pMDC202 vector through BP and LR recombination (Lambda integrase/excisionase; Elpis-Biotech), resulting in the *35S*::*OsSPL10-GFP* plasmid. The fusion construct as well as the control (empty pMDC202 vector; *35S*::*GFP*) were infiltrated into tobacco (*Nicotiana benthamiana*) leaves using a needleless syringe. For agroinfiltration, agrobacteria were grown overnight in Luria–Bertani containing the appropriate antibiotics. The agrobacteria were collected by centrifugation and then re-suspended in 10 mM MgCl_2_ containing 100 mM acetosyringone. After incubated for a minimum of 2 h at room temperature, the culture was diluted to an OD600 of 0.2. Tobacco plants were agroinfiltrated with appropriate agrobacterial cultures, and the agroinfiltrated plants were maintained under normal growth conditions for 12 to 72 h. The DAPI (4’, 6-diamidino-2-phenylindole) was used to confirm nucleus. The tobacco cell layers were examined with a confocal laser scanning microscope. (TCS SP8, Leica, Germany).Data availabilityAll data generated or analyzed during this study are included in this published article.

## Results

### Knockout of OsSPL10 enhances salt tolerance but inhibits trichome development

**Figure 1:**
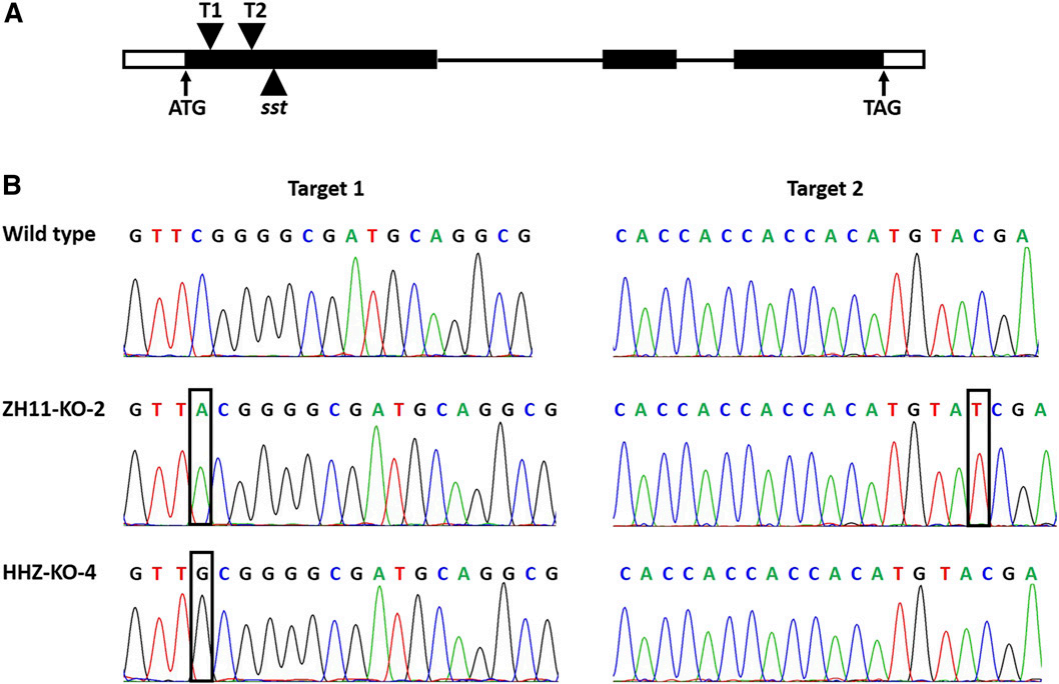
CRISPR/Cas9 editing of *OsSPL10* in ZH11 and HHZ. A The gene framework of *OsSPL10* showing the coding region (filled boxes), UTRs (blank boxes), introns (horizontal lines), positions of *sst* mutation site (filled triangle) and CRISPR/Cas9 target sites (reversed filled triangles). T1, target site 1; T2, target site 2. B The target-site sequences of wild type, ZH11-KO-2 and HHZ-KO-4. The letters in boxes represent inserted nucleotides.

In the experiment of CRISPR/Cas9 editing of *OsSPL10*, 28 and 20 positive transgenic (T_0_) plants were obtained from ZH11 and HHZ, respectively. Among the T_0_ plants, 25 (89.3%) from ZH11 and 15 (75%) from HHZ had mutations at either or both of the target sites, with 7 from ZH11 and 3 from HHZ being homozygous with the mutant allele. Protein sequence analysis predicted that all of the mutations resulted in a premature stop codon. Therefore, the mutants obtained were all *OsSPL10*-knockout mutants (denoted as ZH11-KO and HHZ-KO, respectively). We chose two mutants, one from ZH11 and HHZ each, named ZH11-KO-2 and HHZ-KO-4, respectively (Figure 1), to investigate the effects of *OsSPL10* mutation on salt tolerance and trichome development.

**Figure 2:**
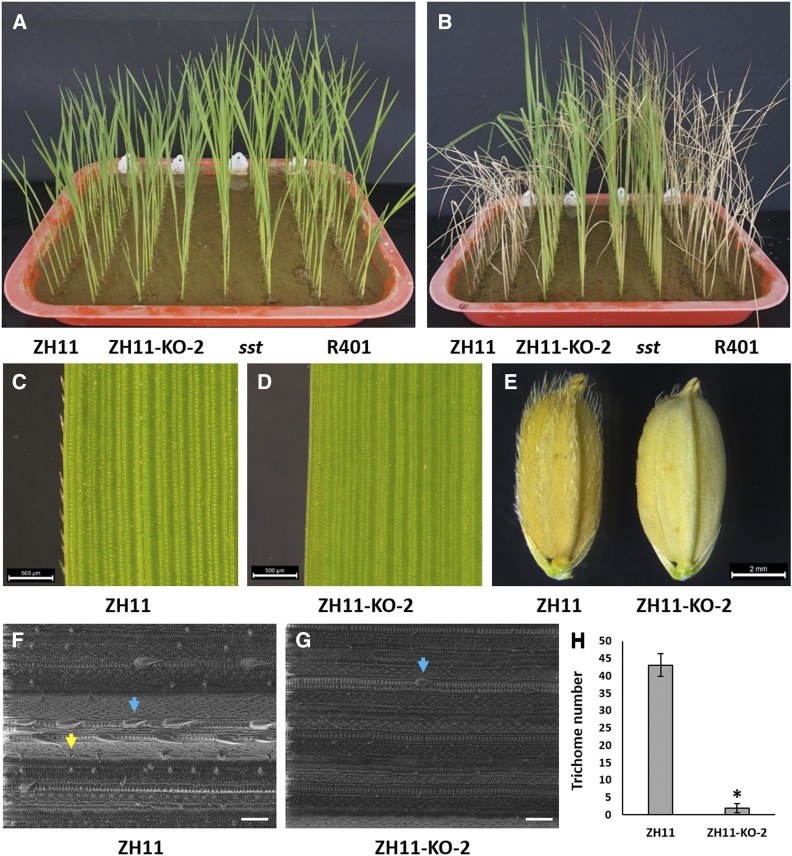
Effects of *OsSPL10* mutation on salt tolerance and trichome development in ZH11. A Ten-day seedlings of ZH11, ZH11-KO-2, *sst* and R401 without salt stress. B Seedlings of ZH11, ZH11-KO-2, *sst* and R401 after salt stress treatment. C–D Mature leaves of ZH11 (C) and ZH11-KO-2 (D) at heading stage. E Seeds of ZH11 and ZH11-KO-2. F–G SEM images of ZH11 (F) and ZH11-KO-2 (G) leaves at heading stage. H Density (number/vision field) of trichomes on leaf in ZH11 and ZH11-KO-2 at heading stage. For each line, the density was calculated based on 6 fields of vision in the SEM images of two penultimate leaves at heading stage. The blue and yellow arrows indicate a macrohair and a microhair, respectively. The asterisks represent significant difference from control ZH11 at *P* < 0.05. Scale bar = 500 μm (C and D), 2 mm (E) and 100 μm (F and G).

**Figure 3:**
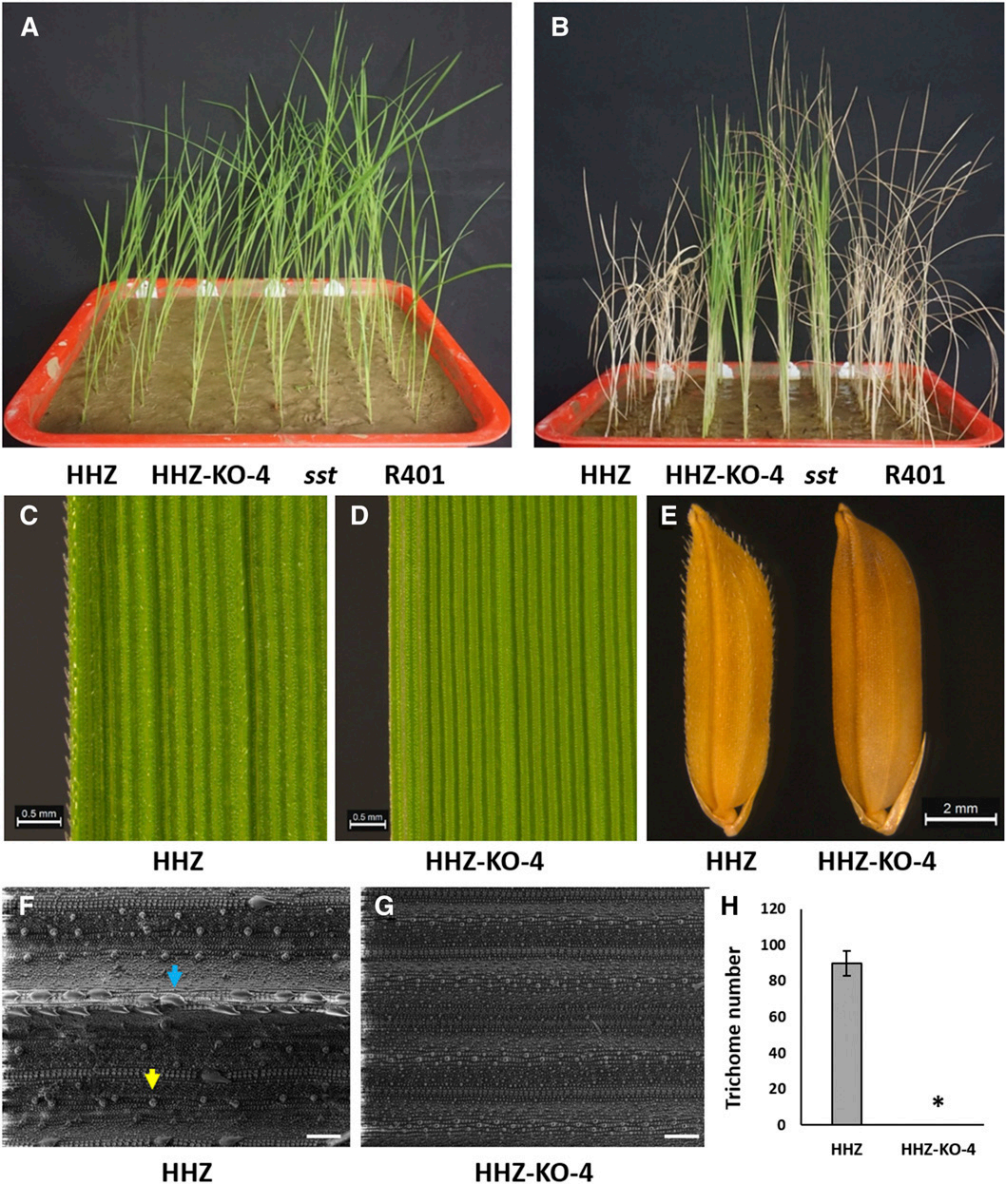
Effects of *OsSPL10* mutation on salt tolerance and trichome development in HHZ. A Twelve-day seedlings of HHZ, HHZ-KO-4, *sst* and R401 without salt stress. B Seedlings of HHZ, HHZ-KO-4, *sst* and R401 after salt stress treatment. C–D Mature leaves of HHZ (C) and HHZ-KO-4 (D) at heading stage. E Seeds of HHZ and HHZ-KO-4. F–G SEM images of HHZ (F) and HHZ-KO-4 (G) leaves at heading stage. H Density (number/vision field) of trichomes on leaf in HHZ and HHZ-KO-4 at heading stage. For each line, the density was calculated based on 6 fields of vision in the SEM images of two penultimate leaves at heading stage. The blue and yellow arrows indicate a macrohair and a microhair, respectively. The asterisks represent significant difference from control HHZ at *P* < 0.05. Scale bar = 500 μm (C and D), 2 mm (E) and 100 μm (F and G).

Both ZH11-KO-2 and HHZ-KO-4 showed significantly higher tolerance to salt stress than their corresponding wild types in the experiment. While all of the ZH11 and HHZ seedlings died (survival rate = 0%) at the end of salt treatment, the ZH11-KO-2 and HHZ-KO-4 seedlings still all kept alive (survival rate = 100%), similar to the case of *sst vs.* R401 (Figure 2A, B and 3A, B). Meanwhile, both ZH11-KO-2 and HHZ-KO-4 displayed glabrous leaves and glumes without or with very few trichomes as expected (Figure 2C-E and 3C-E). SEM observation showed that the trichome density on leaf surface (number of trichomes per vision) at heading stage was ∼43 in ZH11 (Figure 2F and H) and ∼88 in HHZ (Figure 3F and H), respectively, whereas the density was only ∼2 in ZH11-KO-2 (Figure 2G and H) and nearly 0 in HHZ-KO-4 (Figure 3G and H), respectively. These results indicated that loss of *OsSPL10* function can result in higher salt tolerance as well as glabrous leaves and glumes, confirming that *OsSPL10* is *SST*. In addition, the ZH11-KO-2, HHZ-KO-4 and *sst* seedlings all appeared to be a little taller than those of their corresponding wild types (Figure 2A and 3A), suggesting that loss of *OsSPL10* function has an effect of promoting plant growth.

### Overexpression of OsSPL10 reduces salt tolerance but promotes trichome development

**Figure 4:**
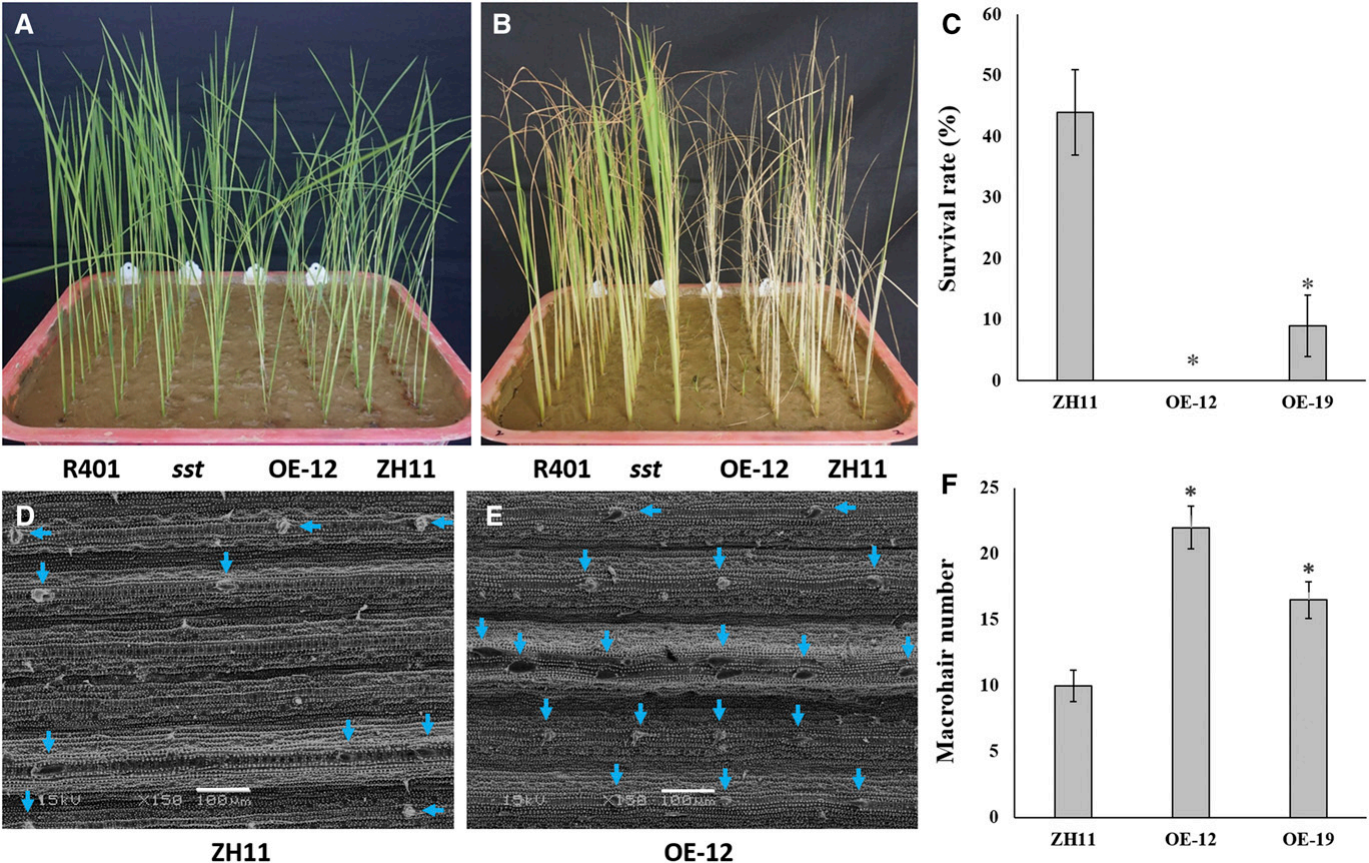
Effects of *OsSPL10* overexpression on salt tolerance and trichome development in ZH11. A Ten-day seedlings of R401, *sst*, ZH11-OE-12 and ZH11 without salt stress. B Seedlings of R401, *sst*, ZH11-OE-12 and ZH11 after salt stress treatment. C Survival rates of ZH11, ZH11-OE-12 and ZH11-OE-19 after salt stress treatment. For each line, the survival rate was calculated based on three replicates (trays) with 22 seedlings in each replicate. D–E SEM images of ZH11 (D) and ZH11-OE-12 (E) leaves at tillering stage. F Density (number/vision field) of macrohairs on leaf in ZH11, ZH11-OE-12 and ZH11-OE-19 at tillering stage. For each line, the density was calculated based on 6 fields of vision in the SEM images of two penultimate leaves at tillering stage. The blue arrows indicate macrohairs. The asterisks represent significant difference from control ZH11 at *P* < 0.05. Scale bar = 100 μm.

A total of 22 positive transgenic (T_0_) plants overexpressing *OsSPL10* were acquired, among which plant ZH11-OE-12 showed the highest level of *OsSPL10* expression, followed by plant ZH11-OE-19. We examined the phenotypes of the stably-inherited homozygous progeny lines of ZH11-OE-12 and ZH11-OE-19. Contrary to the *OsSPL10*-knocked-out seedlings, the *OsSPL10*-overexpressed seedlings were a little shorter (Figure 4A) but more sensitive to salt stress than the wild-type seedlings (Figure 4B). At the end of salt treatment there were still ∼44% ZH11 seedlings alive, while the ZH11-OE-12 seedlings all died and only ∼9% ZH11-OE-19 seedlings survived (Figure 4C). Since *OsSPL10* expression was stronger in ZH11-OE-12 than in ZH11-OE-19, the result suggested that higher *OsSPL10* expression level would lead to higher sensitivity to salt. In addition, SEM observation at tillering stage indicated that the *OsSPL10*-overexpressed plants had higher density of macrohairs on leaf (Figure 4E) than wild type (Figure 4D), and the density also appeared to be positively proportional to the level of *OsSPL10* expression (Figure 4F). These results indicated that *OsSPL10* overexpression had exactly the opposite effect to that of *OsSPL10* knockout, and the effect increased with the increase of *OsSPL10* expression. This validated the function of *OsSPL10* known from its loss-of-function mutants, further confirming that *OsSPL10* is *SST*.

### OsSPL10 is preferentially expressed in young panicle and stem

**Figure 5:**
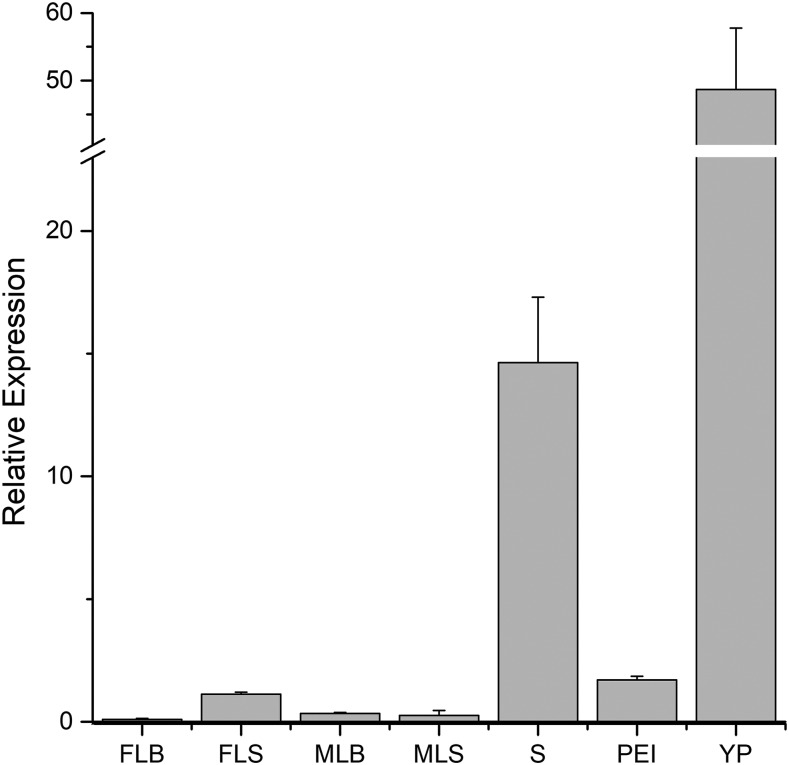
The expression pattern of *OsSPL10* at the booting stage. FLB, flag leaf blade; FLS, flag leaf sheath; MLB, mature leaf blade; MLS, mature leaf sheath; S, stem; PEI, pre-emergence inflorescence; YP, young panicle

To examine the potential tissue specificity of *OsSPL10*, we used qRT-PCR to analyze the expression pattern of *OsSPL10* at the booting stage. We found that *OsSPL10* was preferentially expressed in early young panicles (< 5 cm) and stem, while its expression levels in late young panicles (pre-emergence inflorescence, 5-10 cm), leaf blades and leaf sheaths were generally low or very weak (Figure 5). These results suggested that *OsSPL10* is probably involved in the early development of inflorescence, or in the phase transition from vegetative growth to reproductive development.

### OsSPL10 is localized in nucleus

**Figure 6:**
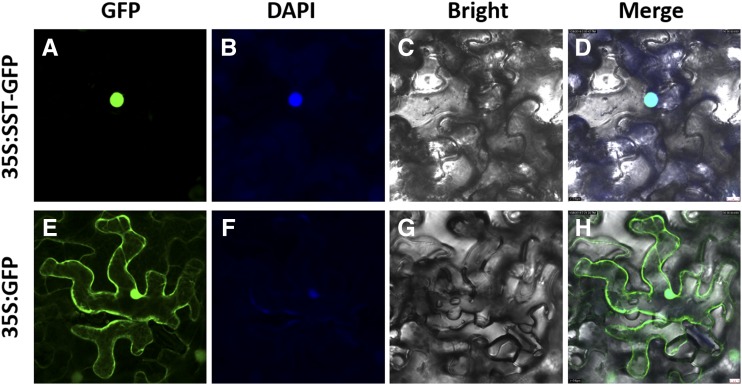
Subcellular localization of OsSPL10-GFP fusion protein. A-D Transient co-expression of fusion protein OsSPL10-GFP and DAPI. E-F Transformation with *35S*::*GFP* as control. Scale bar = 10 μm, applicable to all photos.

Some *SPL* genes playing important roles in growth and development have been found to function as transcription factors (Wang *et al.* 2009; Jiao *et al.* 2010). Therefore, we predicted that OsSPL10 protein might be also a transcription factor, which should be sorted to nucleus. Transient expression of *35S*::*OsSPL10-GFP* in the epidermal cells of *Nicotiana benthamiana* (tobacco) leaves clearly showed that the GFP signal of SST-GFP fusion protein was observed only in nucleus (Figure 6A-D). By contrast, the GFP signal due to transformation of *35S*::*GFP* was observed everywhere in the cell without specificity (Figure 6E-H). These results supported our prediction that SST is localized in the nucleus, suggesting that SST possibly functions as a transcription factor.

## Discussion

In this study, we confirmed through gene knockout and gene overexpression that *OsSPL10* is *SST*, which plays a negative role in salt tolerance but a positive role in trichome formation and has a small negative effect on seedling growth as well in rice. In addition, the result of subcellular localization supported the prediction that OsSPL10 probably functions as a transcription factor like other OsSPL proteins.

There are 19 *OsSPL* genes in rice, including one pseudogene. Among them, 11 genes (not including *OsSPL10*) are the targets of miR156 (Xie *et al.* 2006). *OsSPL10* is the first *OsSPL* gene confirmed to control salt tolerance in rice. However, there could be other *OsSPL* genes related to salt tolerance. It has been found that the abundance of miR156 increases in rice plants when subjected to salt stress, and the transgenic rice seedlings overexpressing miR156 show higher salt tolerance (Cui *et al.* 2014). This implies that there might be some *OsSPL* genes targeted by miR156 negatively controlling salt tolerance in rice. If this is true, there will be two different pathways of *OsSPL*-mediated salt tolerance regulation in rice. One is miR156-dependent, the other is miR156-independent (*e.g.*, mediated by *OsSPL10*). But no matter in what pathways, the *OsSPL* genes involved all function as a negative regulator.

To date, two genes controlling trichome development have been reported in rice, namely, *OsWOX3B* (*DEP*, *NUDA*/*GL-1*, *GLR1*) and *OsPLT2* (*HL6*) (Angeles-Shim *et al.* 2012; Zhang *et al.* 2012; Li *et al.* 2012; Sun *et al.* 2017). *OsWOX3B* belongs to the WOX3 family of plant-specific homeobox transcription factors (Angeles-Shim *et al.* 2012), while *OsPLT2* is an AP2/ERF transcription factor. *OsPLT2* regulates trichome elongation, which is dependent on functional OsWOX3B that acts as a key regulator in trichome initiation (Sun *et al.* 2017). In this study, we found that knockout of *OsSPL10* exhibited glabrous leaves and glumes, while overexpression of *OsSPL10* increased the density of trichomes on leaves. In *Arabidopsis*, *SPLs* have also been found to be involved in the development and distribution of trichomes (Unte *et al.* 2003; Yu *et al.* 2010). The effect of *OsSPL10* on trichome development found in this study is more similar to that of *OsWOX3B* than to that of *OsPLT2*. Therefore, we speculate that *OsSPL10* is likely to control trichome initiation. As an SBP-box gene with its protein being localized in nucleus (Figure 6), *OsSPL10* probably function as transcription factor in trichome initiation. Further research is needed to clarify how *OsSPL10* regulates trichome development and what relationship exists among *OsWOX3B*, *OsPLT2*, and *OsSPL10* in rice.

## Conclusion

*OsSPL10* plays a dual role in salt tolerance and trichome formation in rice. It negatively controls salt tolerance but positively controls trichome initiation. The results of this study will help further research and better understanding of the mechanisms of salt tolerance and trichome formation in rice.
